# Sustained‐release ruxolitinib: Findings from a phase 1 study in healthy subjects and a phase 2 study in patients with myelofibrosis

**DOI:** 10.1002/hon.2544

**Published:** 2018-09-11

**Authors:** Srdan Verstovsek, Swamy Yeleswaram, Kevin Hou, Xuejun Chen, Sue Erickson‐Viitanen

**Affiliations:** ^1^ The University of Texas MD Anderson Cancer Center Houston TX USA; ^2^ Incyte Corporation Wilmington DE USA

**Keywords:** clinical trials, experimental, formulation, oncology, pharmacokinetics

## Abstract

Ruxolitinib is the first Janus kinase (JAK) inhibitor approved for the treatment of myelofibrosis, where its efficacy is often associated with cytopenia. It is possible that the severity of cytopenia is in part driven by *C*_max_. A once‐daily sustained‐release (SR) formulation of ruxolitinib was therefore developed to decrease the *C*_max_/*C*_min_ ratio relative to twice‐daily immediate‐release (IR) ruxolitinib. An SR formulation was identified based on pharmacokinetic evaluation in a phase 1 study in healthy adults (N = 9). This was followed by an open‐label phase 2 study in patients with myelofibrosis (N = 41). Ruxolitinib SR treatment was well tolerated with blood cell counts relatively unchanged through week 16. In terms of efficacy, 7 patients (17.1%) had clinical improvement and 33 (80.5%) had stable disease. While this study has raised the possibility of an increased therapeutic index for ruxolitinib via an SR formulation, further studies are required to validate the hypothesis.

## 1 INTRODUCTION

A twice‐daily (BID) immediate‐release (IR) formulation of ruxolitinib is approved for the treatment of patients with intermediate‐risk or high‐risk myelofibrosis (MF), including primary MF (PMF), postpolycythemia vera MF (PPV‐MF), and postessential thrombocythemia MF (PET‐MF).^1^ Phase 3 randomized clinical trials demonstrated that ruxolitinib IR reduced splenomegaly and improved MF‐related symptoms and quality of life compared with controls.^2^, ^3^ Furthermore, data from these trials strongly suggested that ruxolitinib prolonged survival relative to placebo or best available therapy.^4^, ^5^ However, in some patients, ruxolitinib can cause thrombocytopenia and anemia, typically during the first 8 to 12 weeks of therapy, which may result in dose reductions or treatment discontinuation.^1^

A once‐daily (QD) sustained‐release (SR) formulation of ruxolitinib was developed in an effort to generate an efficacious treatment option while limiting the risk and severity of cytopenias by decreasing the maximal plasma exposure to ruxolitinib. A QD formulation could also provide a more convenient treatment regimen for patients with MF. Across a variety of treatment settings and disease states, patient adherence to prescribed treatment is optimized by limiting the number of required daily doses.^6^, ^7^

This report presents data from 2 sequential studies that evaluated SR formulations of ruxolitinib. A phase 1 study in healthy subjects (INCB 18424‐139) compared the bioavailability of ruxolitinib SR and IR to determine a suitable formulation and dose for evaluation in patients with MF. A phase 2 study (INCB 18424‐260; NCT01340651) subsequently evaluated the pharmacokinetic properties and clinical activity of ruxolitinib SR in patients with MF.

## 2 METHODS

### 2.1 Ethics

Both studies were conducted in accordance with the Declaration of Helsinki and *Good Clinical Practice: Consolidated Guideline* approved by the International Conference on Harmonisation. The respective clinical study protocols, amendments, informed consent documents, and other appropriate study‐related documents were reviewed and approved by independent ethics committees/institutional review boards. Written informed consent was obtained from all participants.

### 2.2 Phase 1 study: healthy subjects

Details about enrolled subjects, study design, study endpoints, and statistical analyses for the phase 1 study of healthy subjects can be found in the Supplemental Appendix.

### 2.3 Phase 2 study: patients with myelofibrosis

#### 2.3.1 Patients

All enrolled participants were adults (aged ≥18 years) diagnosed with PMF, PPV‐MF, or PET‐MF for which treatment was indicated as per physician assessment. Study participants had a life expectancy ≥6 months and a spleen length ≥5 cm below the costal margin (determined by palpation).

#### 2.3.2 Study design and dosing

INCB 18424‐260 was a single‐arm, open‐label phase 2 trial of ruxolitinib SR tablets QD. Study visits occurred at screening; baseline; day 1; weeks 2, 4, 8, 12, 16, 20, and 24; and every 12 weeks thereafter. All patients began treatment with 25 mg ruxolitinib SR QD. After 8 or 12 weeks, the dose level of ruxolitinib could be titrated to 50 mg SR QD for inadequate efficacy. To address potential toxicity arising from the increased dose, the protocol was amended to include an optional titration to ruxolitinib 25 mg SR alternating with 50 mg SR every other day (QOD), depending on platelet count; this amendment occurred after the study began (patients entering the study before the amendment did not have this option). Efficacy was considered inadequate if patients had <40% reduction from baseline in palpable spleen length at the week 8 or 12 study visit.

At week 16, all patients transitioned to ruxolitinib IR BID, with the starting dose depending on platelet count: Patients with a platelet count ≥200 × 10^9^/L initiated 20 mg IR BID, those with a platelet count 100 to <200 × 10^9^/L initiated 15 mg IR BID, and those with a platelet count 75 to <100 × 10^9^/L initiated 10 mg IR BID. All patients with platelet counts between 50 and <75 × 10^9^/L were subject to a mandatory dose reduction to ruxolitinib IR 5 mg BID. Further treatment with ruxolitinib was withheld from patients with platelet counts <50 × 10^9^/L. Doses were restarted or increased after platelet counts or absolute neutrophil count (ANC) levels recovered to acceptable levels. Patients had the option to remain on ruxolitinib IR treatment until ruxolitinib IR tablets became commercially available or until the last patient completed 36 weeks of treatment, whichever occurred earlier.

#### 2.3.3 Study endpoints

The primary study endpoints were safety/tolerability and overall response (OR). Safety/tolerability assessments included monitoring adverse events (AEs), vital signs, and clinical laboratory data. Changes from baseline in platelet count over time and the proportion of patients with grade 3/4 thrombocytopenia were also analyzed. To assess OR, the proportion of patients with International Working Group‐Myelofibrosis Research and Treatment (IWG‐MRT) responses^8^ was analyzed at each study visit and designated as clinical response, stable disease, or progressive disease. Although the IWG‐MRT criteria include responses designated as complete remission (CR) and partial remission (PR), postbaseline bone marrow samples were not collected to confirm CR versus PR.

Secondary endpoints included changes from baseline in spleen volume, Modified Myelofibrosis Symptom Assessment Form (MFSAF) Total Symptom Score (TSS), and pharmacokinetic assessments. Changes from baseline in spleen volume were measured by magnetic resonance imaging, and spleen length was measured by palpation. The proportion of patients with a ≥35% reduction in spleen volume from baseline and a ≥50% reduction in MFSAF TSS from baseline were assessed at week 16. Assessed pharmacokinetic parameters included minimum plasma concentration (*C*_min_), maximum plasma concentration (*C*_max_), time to *C*_max_ (*t*_max_), area under the concentration time curve (AUC) from time 0 to 24 hours (steady state), AUC from time 0 to last measurable concentration (AUC_0‐t_) (single dose), terminal elimination half‐life (*t*_½_), oral‐dose clearance (CL/F), and oral‐dose volume of distribution (V_z_/F).

#### 2.3.4 Statistical analyses

Descriptive summaries were included for continuous and categorical variables. Unless otherwise stated, all CIs were 2‐sided 95% CIs, unadjusted for multiplicity. The safety population was used for all safety analyses; the intent‐to‐treat population was used for efficacy analyses. Pharmacokinetic data were analyzed by population pharmacokinetic analysis using pharmacokinetic‐evaluable subjects.

## 3 RESULTS

### 3.1 Phase 1 study: healthy *s*ubjects

#### 3.1.1 Subject disposition and demographics

Nine healthy adults (6 men and 3 women) were enrolled in the phase 1 study and received 25 mg ruxolitinib IR. Eight of the 9 subjects remained on‐study to receive treatment with 2 different ruxolitinib SR formulations (SR‐1 and SR‐2). The median (range) age of study participants was 27 (18‐53) years. Eight subjects (88.9%) were white; 1 (11.1%) was African American.

#### 3.1.2 Safety

No deaths or serious AEs (SAEs) occurred during this study. Elevated blood creatine phosphokinase led to study withdrawal in 1 subject after administration of ruxolitinib IR. This treatment‐emergent AE (TEAE) was classified as moderate in intensity and was considered unrelated to study medication. Overall, safety was similar to that previously observed with ruxolitinib IR in healthy subjects.^9^

#### 3.1.3 Pharmacokinetics

**Figure 1:**
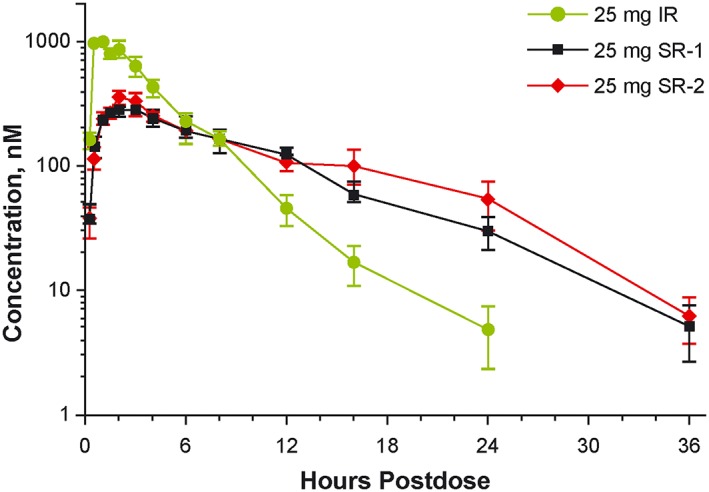
Phase 1 study of healthy subjects: mean plasma concentrations in fasted subjects receiving single doses of ruxolitinib 25 mg as IR (n = 9), SR‐1 (n = 8), and SR‐2 (n = 8) formulations. IR, immediate release; SR, sustained release. Data are mean ± SE

Plasma concentrations of ruxolitinib IR and SR over time after administration in fasted subjects are presented in Figure 1. The mean *C*_max_ was 1100 nM for the IR formulation, compared with 333 nM for SR‐1 and 394 nM for SR‐2 (Table 1). These values represent reductions in *C*_max_ of 69.7% and 64.2%, respectively, versus the IR formulation.

**Table 1:** Phase 1 study of healthy subjects: pharmacokinetic parameters

Parameter	Ruxolitinib, Mean (SD)			*P* Value^b^	Bioavailability, % (90% CI)^a^	
	IR (n = 9)	SR‐1 (n = 8)	SR‐2 (n = 8)		SR‐1 vs IR	SR‐2 vs IR
*C*_max_, nM	1100 (332)	333 (76.1)	394 (126)	<.0001	30.4 (25.4‐36.4)	35.2 (29.5‐42.2)
*t*_max_, h	0.9 (0.5)	2.4 (1.0)	2.9 (1.6)	.0003	NA	NA
*C*_12h_, nM	45.6 (38.1)	121 (46.8)	104 (43.2)	NA	NA	NA
*C*_max_/C_12h_	40 (24)	3.0 (1.0)	4.7 (3.1)	NA	NA	NA
*t*_½_, h	2.8 (0.7)	5.3 (1.8)	6.1 (2.1)	<.0001	NA	NA
AUC_0‐t_, nM·h	4340 (1990)	3110 (840)	3520 (1260)	.040	74.7 (62.2‐89.7)	82.5 (68.7‐99.1)
AUC_0‐∞_, nM·h	4350 (1990)	3180 (864)	3740 (1400)	.070	76.2 (63.1‐92.0)	86.7 (71.8‐105)
CL/F, L/h	22.8 (10.3)	27.2 (6.7)	24.6 (9.0)	.070	NA	NA

Abbreviations: AUC_0‐t_, area under the concentration‐time curve from time 0 to last measurable concentration; AUC_0‐∞_, area under the concentration‐time curve from time 0 extrapolated to infinity; *C*_max_, maximum plasma concentration; *C*_12h_, plasma concentration at 12 hours after dosing; *C*_max_/*C*_12h_, ratio of the maximum plasma concentration to the plasma concentration at 12 hours after dosing; CL/F, oral dose clearance; IR, immediate release; NA, not applicable; SR, sustained release; *t*_½_, elimination half‐life; *t*_max_, time to maximum plasma concentration.

a Geometric mean relative bioavailability.

b *P* values from a crossover analysis of variance of log‐transformed data.

The *t*_½_ of ruxolitinib approximately doubled for both SR formulations compared with the IR formulation (Table 1). The ratio of *C*_max_ to plasma concentration at 12 hours after dosing (*C*_12h_) decreased from 40 for ruxolitinib IR to 3.0 for ruxolitinib SR‐1 and 4.7 for SR‐2. Compared with ruxolitinib IR, the relative bioavailability of ruxolitinib SR‐1 and SR‐2 was 76% and 87%, respectively.

Pharmacokinetic simulation indicated that ruxolitinib SR‐1 and SR‐2 could provide higher steady‐state mean *C*_min_ values compared with ruxolitinib IR (6.5‐fold and 11.4‐fold, respectively) and lower steady‐state mean *C*_max_ values (69% and 61%). The SR‐2 formulation was ultimately selected for further development because of a slightly higher relative bioavailability compared with the SR‐1 formulation (Table 1).

### 3.2 Phase 2 study: Patients *w*ith myelofibrosis

#### 3.2.1 Patient disposition and characteristics

For weeks 1 to 16 of the phase 2 study, patients received 25 mg ruxolitinib SR QD; at week 16, all patients transitioned to 25 mg ruxolitinib IR BID. Of the 41 patients enrolled, 39 (95.1%) completed through week 16, and 24 (58.5%) completed through week 24. Reasons for study withdrawal before week 16 included AEs (n = 1) and transferring to treatment with a commercial product (n = 1). Reasons for withdrawal from weeks 16 to 24 included transferring to treatment with a commercial product (n = 11), consent withdrawn (n = 2), and AEs (n = 2).

Baseline demographics, disease characteristics, and laboratory values are presented in Table 2. Forty‐one adult patients (median [range] age, 68 [50‐81] years) with PMF (78.0%), PPV‐MF (17.1%), or PET‐MF (4.9%) enrolled in this study. A majority of patients were men (65.9%) and were white (97.6%).

**Table 2:** Phase 2 study of patients with myelofibrosis: baseline demographics and disease characteristics, intent‐to‐treat population

Characteristic	Ruxolitinib (N = 41)
Median (range) age, years	68.0 (50.0‐81.0)
Sex, n (%)	
Male	27 (65.9)
Female	14 (34.1)
Race, n (%)	
White	40 (97.6)
Asian	1 (2.4)
IPSS risk category, n (%)	
High, ≥3 factors	19 (46.3)
Intermediate, 2 factors	22 (53.7)
ECOG performance status, n (%)	
0	3 (7.3)
1	26 (63.4)
2	11 (26.8)
3	1 (2.4)
MF subtype, n (%)	
Primary	32 (78.0)
Post‐polycythemia vera	7 (17.1)
Post‐essential thrombocythemia	2 (4.9)
Prior hydroxyurea use, n (%)	
Yes	18 (43.9)
No	23 (56.1)
Transfusion status, n (%)	
Independent	38 (92.7)
Dependent	3 (7.3)
Median (range) palpable spleen size below costal margin, cm	18.0 (7.0‐30.0)
Median (range) spleen volume, cm^3^ a	2592.7 (697.1‐5926.1)
Mean (SD) total symptom score	21.4 (12.03)
*JAK2* mutation, n (%)	
Positive	25 (61.0)
Negative	7 (17.1)
Missing	9 (22.0)
Median (range) percentage V617F of positive history *JAK2* mutation^b^	79.0 (44.0‐96.0)
Median (range) baseline laboratory values, ×10^9^/L^a^	
Platelet count	217.5 (108.0‐1030.0)
Hemoglobin	107.0 (68.0‐135.0)
Absolute neutrophil count	12.5 (1.3‐35.3)
Lymphocyte count	1.3 (0.21‐4.54)

Abbreviations: ECOG, Eastern Cooperative Oncology Group; IPSS, International Prognostic Scoring System; MF, myelofibrosis.

a n *=* 40.

b n = 25.

Overall, 38 patients (92.7%) were transfusion‐independent and 22 (53.7%) were designated intermediate‐2 risk according to International Prognostic Scoring System risk category^10^; 19 (46.3%) were designated high risk. A *Janus kinase* (*JAK*) *2* mutation was present in 61.0% of patients, and the median spleen volume at baseline was 2592.7 cm^3^, which is consistent with massive splenomegaly.

For most patients, platelet counts were within normal limits at baseline. The median hemoglobin level of patients was below the lower limit of normal (130 × 10^9^/L for men and 120 × 10^9^/L for women); 37 (90.2%) had decreased hemoglobin of at least grade 1 at baseline, and 15 (36.6%) had grade 2 low hemoglobin levels (ie, <100 × 10^9^/L). The median ANC was above the upper limit of normal (7.9 × 10^9^/L).

#### 3.2.2 Safety/tolerability

All enrolled patients were in the safety‐evaluable population (n = 41). Through week 16, the median duration of exposure to ruxolitinib SR was 113.0 days, with an average daily dose of 26.1 mg.

Through week 16, 32 of 41 patients (78.0%) receiving ruxolitinib SR experienced a TEAE. The most common TEAEs through week 16 were diarrhea (19.5%), peripheral edema (17.1%), and thrombocytopenia/decreased platelet count (17.1%). Other TEAEs occurring in ≥5% of patients were bone pain (9.8%) and anemia, asthenia, nausea, pruritus, and sinusitis (7.3% each). A grade ≥3 TEAE was observed in 7 of 41 patients (17.1%), including 1 patient (2.4%) who experienced grade 3 thrombocytopenia. Treatment‐emergent cytopenias occurring after week 16 included thrombocytopenia (4.9%) and anemia (2.4%).

Through week 16, 1 patient (2.4%) experienced TEAEs leading to study withdrawal (day 8 gastritis and gastrointestinal hemorrhage [neither related to study treatment] with a platelet count of 305 × 10^9^/L). The gastrointestinal hemorrhage resolved following hospitalization (gastritis was ongoing). After week 16, 2 patients (4.9%) withdrew due to TEAEs (anemia, n = 1; cardiac arrest, n = 1).

**Figure 2:**
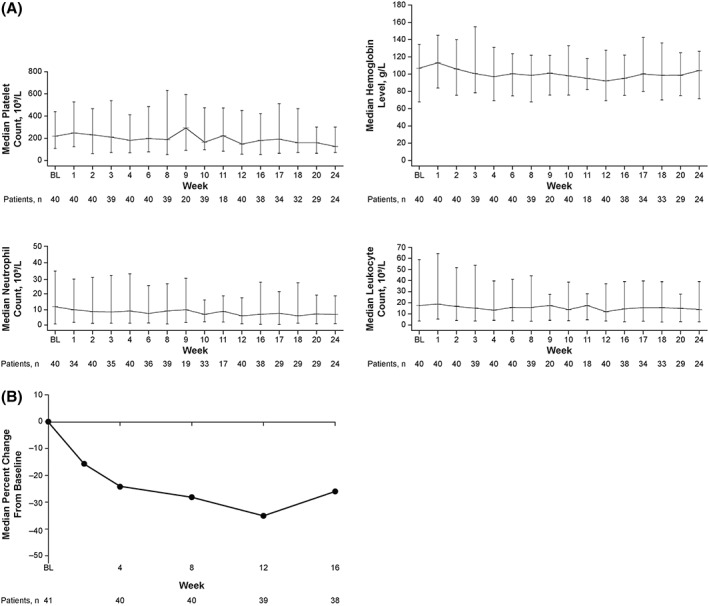
Phase 2 study of patients with myelofibrosis: blood counts over time (A)^a^ and median percentage change in spleen length from baseline to week 16 (B). BL, baseline. ^a^Data are median ± range

The median levels of platelets, hemoglobin, neutrophils, and leukocytes over time are shown in Figure 2A. Most patients (56.1%) maintained their baseline platelet count grade through week 16. Baseline hemoglobin levels were generally maintained throughout the study. Most patients (95.1%) had neutrophil counts in the normal range at baseline, and 92.3% of these patients maintained normal counts through week 16. Similarly, 95.1% of patients had leukocyte counts in the normal range at baseline, and 84.6% maintained normal counts through week 16.

From study initiation through week 16, 6 patients (14.6%) had SAEs; diarrhea and pneumonia were the most common (n = 2 [4.9%] each), while clostridium difficile colitis, dehydration, gastroenteritis, gastrointestinal hemorrhage, generalized edema, hip fracture, hypotension, patella fracture, and pulmonary embolism occurred in 1 patient each (2.4%). None of the SAEs observed through week 16 were deemed related to study treatment. Two patients (4.9%) died after week 16 while receiving ruxolitinib IR; 1 patient died of cardiac arrest, and 1 patient progressed to acute myeloid leukemia. The investigators deemed these events unrelated to ruxolitinib treatment.

#### 3.2.3 Week 16 efficacy

Efficacy endpoints at week 16 are summarized in Table 3. From baseline to week 16, 7 patients (17.1%) had clinical improvement, 33 (80.5%) had stable disease, and 1 (2.4%) had progressive disease.

**Table 3:** Phase 2 study of patients with myelofibrosis: week 16 efficacy endpoints

Endpoint	Ruxolitinib (N = 41)
Overall response, n (%)	
Clinical improvement	7 (17.1)
Stable disease	33 (80.5)
Progressive disease	1 (2.4)
Spleen outcomes	
Median (range) change from BL in spleen volume, %	−21.7 (−64.6 to 43.6)
Patients with ≥35% reduction from BL in spleen volume, n (%)	11 (26.8)
Median (range) reduction from BL in spleen length, %	−25.8 (−100 to 50.0)
Symptoms	
Median (range) change from BL in MFSAF TSS, %	−48.6 (−100 to 12.7)
Patients with ≥50% reduction from BL in MFSAF TSS, n (%)	18 (43.9)

Abbreviations: BL, baseline; MFSAF, Modified Myelofibrosis Symptom Assessment Form; TSS, total symptom score.

At week 16, the median (range) percentage change from baseline in spleen volume was −21.7% (−64.6% to 43.6%), and 11 patients (26.8%) had a ≥35% reduction in spleen volume from baseline. Changes from baseline in palpable spleen length decreased steadily through week 12 (Figure 2B); the median (range) percentage change from baseline to week 16 was −25.8% (−100% to 50.0%).

The median (range) percentage change in MFSAF TSS from baseline to week 16 was −48.6% (−100% to 12.7%); 18 patients (43.9%) had a ≥50% reduction from baseline at week 16.

#### 3.2.4 Pharmacokinetics

At weeks 4 and 12, the median *t*_max_ after the first dose of ruxolitinib was 2.0 hours (Table 4). Plasma concentrations of ruxolitinib declined in a multiphasic fashion (geometric mean terminal‐phase disposition *t*_½_, 5.44‐8.90 hours). Geometric mean CL/F ranged from 20.6 to 29.6 L/hour, and geometric mean V_z_/F ranged from 226 to 305 L.

**Table 4:** Phase 2 study of patients with myelofibrosis: sustained‐release ruxolitinib pharmacokinetic parameters at weeks 4 and 12

	Ruxolitinib 25 mg SR QD		Ruxolitinib 50 mg SR QD	Ruxolitinib 25 mg SR QOD^a^
Parameters, Mean ± SD (Geometric Mean)	Week 4 (n = 36)	Week 12 (n = 18)	Week 12 (n = 11)	Week 12 (n = 5)
*C*_min_, nM	32.9 ± 42.0 (NC)	59.1 ± 49.3 (NC)	70.0 ± 79.4 (NC)	51.3 ± 94.9 (NC)
*C*_max_, nM	411 ± 221 (371)	464 ± 223 (419)	730 ± 334 (664)	407 ± 142 (387)
PT ratio	40.1 ± 55.4 (20.4)	11.7 ± 9.21 (9.30)	28.5 ± 37.5 (15.8)	45.9 ± 63.4 (16.84)
*t*_½_, h	7.21 ± 3.67 (6.37)	8.46 ± 3.62 (7.66)	6.12 ± 2.98 (5.44)	16.3 ± 17.8 (8.90)
AUC_t_, nM·h	2110 ± 1260 (1830)	2590 ± 1320 (2290)	4260 ± 1730 (3880)	2370 ± 1060 (2180)
AUC_0‐t_, nM·h	3320 ± 2280 (2750)	4520 ± 2390 (3960)	6670 ± 3410 (5870)	4010 ± 2460 (3440)
CL/F, L/h	34.8 ± 18.7 (29.6)	23.6 ± 12.9 (20.6)	32.2 ± 20.2 (27.8)	27.5 ± 15.6 (23.4)
V_z_/F, L	350 ± 212 (289)	287 ± 190 (237)	255 ± 156 (226)	470 ± 540 (305)
Median (range)				
*t*_max_, h	2.0 (1.0‐6.0)	2.0 (0.5‐8.0)	3.0 (1.0‐8.0)	2.0 (0.5‐3.0)

At week 4, there were 36 patients in the 25 mg SR QD group, 2 in the 5 mg IR BID group, and 2 who were not included (1 because of withdrawal from the study and 1 because of a dose interruption). At week 12, there were 18 patients in the 25 mg SR QD group, 11 in the 50 mg SR QD group, 7 in the 25/50 mg QOD group (5 patients took 25 mg SR and 2 took 50 mg SR that day), and 2 in the 5 mg IR BID group.

Abbreviations: AUC_t_, area under the concentration‐time curve at the last measurable concentration; AUC_0‐t_, area under the concentration‐time curve from time 0 to the last measurable concentration; BID, twice daily; *C*_max_, maximum plasma concentration; *C*_min_, minimum plasma concentration; CL/F, oral dose clearance; IR, immediate release; NC, not calculated; PT, peak‐trough; QD, once daily; QOD, every other day; SR, sustained release; *t*_max_, time to maximum plasma concentration; *t*_½_, elimination half‐life; V_z_/F, volume of distribution.

a The 25 mg SR QOD regimen was 25/50 mg SR QOD with the 25 mg dose on the day of plasma concentration collection. The 25/50 mg QOD group that received 50 mg on the pharmacokinetic sampling day (n = 2) was not included in the table because of the small number of patients in this group.

**Figure 3:**
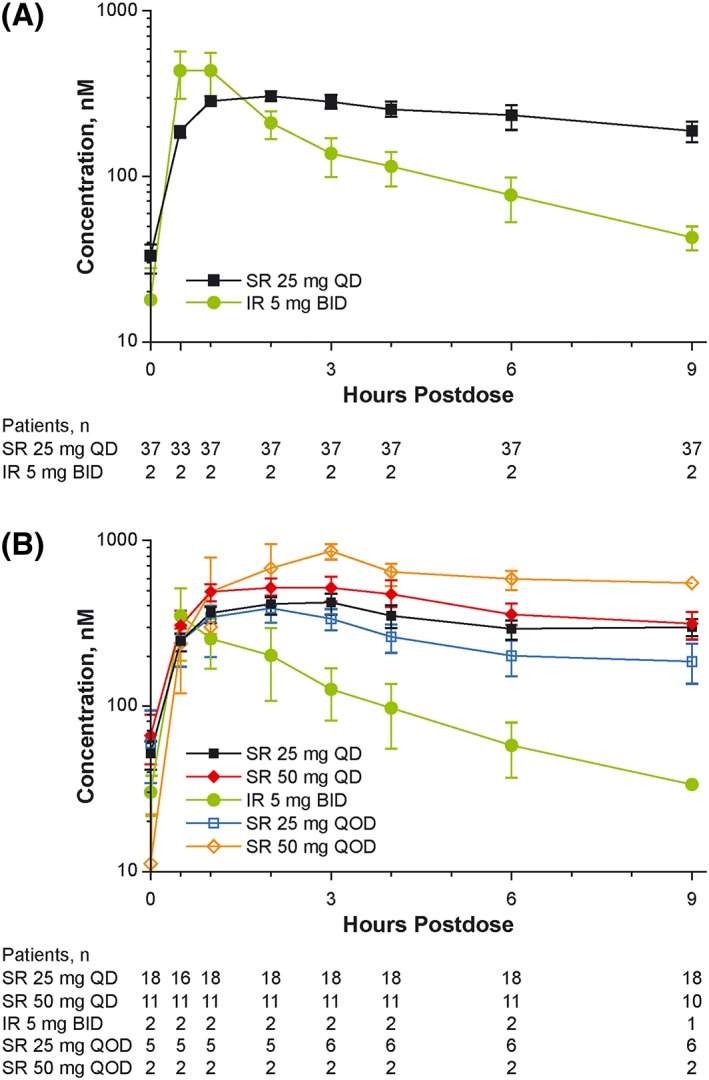
Phase 2 study of patients with myelofibrosis: steady‐state plasma concentrations of ruxolitinib at weeks 4 (A) and 12 (B). BID, twice daily; IR, immediate release; QD, once daily; QOD, every other day; SR, sustained release. Data are mean ± SE. Two patients were not included (1 because of withdrawal from the study and 1 because of a dose interruption)

Steady‐state plasma concentrations of ruxolitinib at weeks 4 and 12 are shown in Figure 3. For ruxolitinib doses of 25 to 50 mg SR QD, the mean steady‐state *C*_max_ and AUC values increased less than proportionally to dose. The geometric mean peak‐to‐trough ratios for SR ranged from 9.3 (week 12; 25 mg SR QD) to 20.4 (week 4; 25 mg SR QD), which exceeded the estimated value of 6.8 based on the pharmacokinetic simulation from the phase 1 study of healthy subjects.

Sex‐based differences were noted in the clearance and volume of distribution of ruxolitinib SR; women had lower CL/F (18.3 vs 34.8 L/hour) and apparent oral‐dose V_z_/F (218 vs 304 L) than men. A sex‐based difference was also observed with ruxolitinib IR (1.25‐fold reduction of CL/F in women vs men).

## 4 DISCUSSION

Pharmacokinetic simulations based on the single‐dose phase 1 study results in healthy subjects demonstrated higher steady‐state mean *C*_min_ values and lower steady‐state mean *C*_max_ values with ruxolitinib SR formulations compared with ruxolitinib IR. In fasted subjects, ruxolitinib SR provided extended absorption of ruxolitinib, with lower maximal exposure compared with ruxolitinib IR without compromising relative oral bioavailability. The ruxolitinib SR‐2 formulation was selected for further development because of a slightly higher relative bioavailability of 87% compared with SR‐1.

In the phase 2 study of patients with MF, the pharmacokinetic characteristics of ruxolitinib SR were similar to those observed in the phase 1 study of healthy subjects. Ruxolitinib SR clearance and volume of distribution were lower in women compared with men. Although smaller in magnitude than was observed with ruxolitinib SR, a similar difference was also observed between women and men receiving ruxolitinib IR. The reason for this difference remains unclear; however, it could not be explained by differences in body weight alone. No other covariates were significant predictors for pharmacokinetic parameters.

Patients receiving ruxolitinib SR exhibited reductions in spleen size compared with baseline, as well as improvement in symptoms as assessed by the MFSAF version 2.0. Ruxolitinib SR was associated with stable hemoglobin and platelet levels, with decreased rates of thrombocytopenia and anemia compared to ruxolitinib IR; safety results were otherwise similar to those reported previously for ruxolitinib IR in patients with MF.^4^, ^5^

Improvements in spleen size with ruxolitinib SR at week 16 were smaller than those reported for ruxolitinib IR in the pivotal COMFORT studies of patients with MF.^2^, ^3^ The proportion of patients treated with ruxolitinib who had a ≥35% reduction in spleen volume from baseline was 26.8% at week 16 in the current phase 2 study compared with 41.9% at week 24 in COMFORT‐I (primary study endpoint) and 32.0% at week 24 in COMFORT‐II (key secondary endpoint).^2^, ^3^ The percentage of patients in the phase 2 study who achieved a ≥50% reduction from baseline in MFSAF TSS at week 16 (43.9%) was similar to that observed at week 24 in COMFORT‐I (secondary endpoint) among patients who received treatment with ruxolitinib (45.9%)^2^; COMFORT‐II did not use the MFSAF for evaluating symptoms.

Care should be taken when viewing these findings in light of important differences between this phase 2 trial of ruxolitinib SR and the phase 3 COMFORT studies of ruxolitinib IR versus placebo^2^ or best available therapy.^3^ For example, our single‐arm, open‐label study enrolled far fewer patients (N = 41) than the randomized COMFORT‐I (N = 309) and COMFORT‐II (N = 219) studies.^2^, ^3^ The COMFORT studies also used dose titration increments of 5 mg,^2^, ^3^ which was not feasible in the phase 2 study of ruxolitinib SR because only 25 mg tablets were available. The timing of measurements also differed between the studies: The primary endpoint and symptom assessments were measured at week 16 here compared with week 24 or weeks 24/48 in the COMFORT‐I and COMFORT‐II studies, respectively.^2^, ^3^

Because ruxolitinib is a balanced JAK1/JAK2 inhibitor, it is unlikely that any potential differences in efficacy between the SR and IR formulations were attributable to JAK enzyme specificity. The variance is most likely due to the differences in peak, average, and minimum inhibition of JAK/signal transducer and activator of transcription signaling. However, an evaluation of isoform specificity was beyond the scope of our analysis.

## 5 STUDY LIMITATIONS

Given the small size of the phase 2 study population, comparisons of efficacy between dose groups could not be adequately assessed. Direct comparisons of the SR and IR formulations were not planned in this phase 2 proof‐of‐concept study, and post hoc comparisons are not feasible because the SR and IR formulations were administered sequentially without a washout period. In addition, several patients dropped out of the study after the week 16 visit before ruxolitinib IR was initiated.

A QOD dosing regimen that was available to some patients in the phase 2 study (25 mg SR QOD/50 mg SR QOD) was not available to the first several patients in the study. As such, dose titration was inconsistent and generally more challenging for the first few patients. Limited dosing options (ie, use of 1 25 mg tablet size) may have precluded effective dose titration and achievement of full clinical benefit for some patients. Finally, the phase 2 study was potentially limited by the exclusion of patients who were intolerant of ruxolitinib IR but may have derived clinical benefit from ruxolitinib SR.

## 6 CONCLUSIONS

The current study results suggest that the higher peak inhibitions achieved with ruxolitinib IR may have incremental contributions to efficacy compared with ruxolitinib SR. However, there was a trend toward a lesser anemia effect with ruxolitinib SR, which could be beneficial for some patients with MF. Collectively, these data suggest that the development of effective SR formulations of ruxolitinib for treating patients with MF is feasible. However, further long‐term studies with multiple ruxolitinib SR dosage strengths will be required to adequately compare efficacy and safety outcomes to those observed with ruxolitinib IR.

## AUTHOR CONTRIBUTIONS

All authors participated in writing the manuscript and analyzing the study data. SV, SY, and SE‐V participated in designing the study research. SV participated in performing the study research.

## FUNDING

This work was funded by Incyte Corporation. Medical writing assistance was provided by Tania Iqbal, PhD (Complete Healthcare Communications, LLC, a CHC Group company), and was funded by Incyte Corporation.

## CONFLICT OF INTEREST/DISCLOSURE

SV participated in advisory boards for Incyte Corporation and received research support for the conduct of clinical studies from Incyte Corporation, Roche, AstraZeneca, Lilly Oncology, Geron, NS Pharma, Bristol‐Myers Squibb, Celgene, Gilead, Seattle Genetics, Promedior, CTI BioPharma Corp, Galena BioPharma, Pfizer, and Genentech. SY, KH, XC, and SE‐V are employees and stockholders of Incyte Corporation.

## Supporting information

Data S1 Supporting Information
