# Supplementary material for: Sustained‐release ruxolitinib: Findings from a phase 1 study in healthy subjects and a phase 2 study in patients with myelofibrosis
Source: Hematol Oncol. 2018 Sep 11;36(4):701–8. doi: 10.1002/hon.2544 (PMC6221065; doi:10.1002/hon.2544)
Supplement: Supplementary file 1 — Data S1 Supporting Information [file HON-36-701-s001.docx]

**Supplemental Appendix**

*Methods ‒ Phase 1 Study: Healthy Subjects*

Subjects

Enrolled subjects were healthy adults (aged 18–55 years) with no clinically significant findings from the screening evaluations (ie, clinical or laboratory tests, and electrocardiograms [ECGs]). All subjects had a body mass index of 18 to 30 kg/m^2^. Women had a negative pregnancy test result at screening and check-in; all subjects agreed to take precautions to avoid pregnancy during the study. Subjects were excluded if they had a history or clinical manifestations of significant metabolic, hepatic, renal, hematologic, pulmonary, cardiovascular, gastrointestinal, urologic, neurologic, or psychiatric disorders.

Study Design and Dosing

INCB 18424-139 was an open-label, single-center, randomized phase 1 trial of ruxolitinib SR compared with ruxolitinib IR. The study design included screening, treatment, and posttreatment study phases.

Subjects were assessed for study eligibility during the screening phase (between Day –28 and Day –2). Subjects were randomly assigned to 3 weekly 1-day treatment periods and received ruxolitinib IR tablets in Period 1 and ruxolitinib SR-1 or SR-2 in Periods 2 and 3, all as single oral 25-mg doses after an overnight fast of ≥10 hours.

The SR formulations were designed to have different dissolution profiles, resulting from the differing amounts and grades of hydroxypropyl methylcellulose. Ruxolitinib SR-1 was designed to be the faster SR tablet formulation; SR-2 was designed to be the slower formulation.

Study medication was administered on Days 1, 8, and 15 of the treatment period (separated by a 5- to 7-day washout period). Serial pharmacokinetic blood samples were collected before study drug administration and at 0.25, 0.5, 1, 1.5, 2, 3, 4, 6, 8, 12, 16, 24, and 36 hours after to determine plasma ruxolitinib concentrations. The pharmacokinetic data from Period 1 were reviewed before selecting SR-1 or SR-2 for evaluation during Periods 2 and 3. The posttreatment phase included a follow-up visit on study Day 29 ±3 days.

Study Endpoints

The primary pharmacokinetic endpoints were C_max_, t_max_, AUC from time 0 to
the last measurable concentration (AUC_0–t_), and AUC from time 0 extrapolated to infinity (AUC_0–∞_). Secondary endpoints included additional pharmacokinetic parameters (C_12h_, C_max_/C_12h_ ratio, t_½_, and CL/F), as well as safety assessments (incidence of adverse events and assessments of ECGs, vital signs, and clinical laboratory evaluations).

Statistical Analyses

Descriptive summaries were included for continuous and categorical variables. The safety population was used for all safety analyses. The threshold for statistical significance was defined as *P*<0.05 (uncorrected *P*, 2-sided statistical tests). Log-transformed pharmacokinetic parameters were compared among treatments using a 2-factor analysis of variance (ANOVA), with the fixed factor for treatment and random factor for subject. The relative bioavailability of the fasted administration of SR formulations versus IR tablets was estimated using the geometric mean relative bioavailability and 90% CI for C_max_, AUC_0-t_, and AUC_0-∞_, which were calculated from the adjusted least squares mean from the ANOVA.
